# Exploring Antibacterial Properties of Mechanochemically Synthesized MgAl_2_O_4_ Spinel Nanoparticles for Dental and Medical Applications

**DOI:** 10.3390/ijms27010438

**Published:** 2025-12-31

**Authors:** Alejandro L. Vega Jiménez, Adriana-Patricia Rodríguez-Hernández, América R. Vázquez-Olmos, Roberto E. Luna-Ramírez, Roberto Y. Sato-Berrú, Roxana Marisol Calderón-Olvera

**Affiliations:** 1 Tissue Bioengineering Laboratory, Division of Postgraduate Studies and Research, Faculty of Dentistry, National Autonomous University of Mexico UNAM, Mexico City 04510, Mexico; robertoerickluna@gmail.com; 2 Molecular Genetics Laboratory, Division of Graduate Studies and Research, Faculty of Dentistry, National Autonomous University of Mexico UNAM, Mexico City 04510, Mexico; aprh_gm@fo.odonto.unam.mx; 3 Hybrid Systems and Nanospectroscopy, Institute of Applied Sciences and Technology, National Autonomous University of Mexico UNAM, Mexico City 04510, Mexico; america.vazquez@icat.unam.mx (A.R.V.-O.); roberto.sato@icat.unam.mx (R.Y.S.-B.); 4 Department of Metallic and Ceramic Materials, Institute of Materials Research, National Autonomous University of Mexico UNAM, Mexico City 04510, Mexico; rcalderon@materiales.unam.mx

**Keywords:** MgAl_2_O_4_, spinel, nanoparticles, antibacterial, mechanosynthesis

## Abstract

Magnesium aluminate spinel nanoparticles (MgAl_2_O_4_-S-NPs) represent a promising class of nanoceramics with potential biomedical applications due to their physicochemical stability and antimicrobial properties. This study aimed to determine the structural characteristics, composition, and biological performance of MgAl_2_O_4_ spinel nanoparticles that were synthesized via a mechanochemical method. Structural and compositional characterization was performed using X-ray diffraction (XRD) and high-resolution transmission electron microscopy (HR-TEM). Antibacterial activity was evaluated against *Helicobacter pylori* and *Enterococcus faecalis* using bacterial viability assays. Structural and morphological analyses confirmed the successful formation of single-phase cubic MgAl_2_O_4_ with a polyhedral morphology and nanoscale size distribution. Bacterial viability was quantified through optical density measurements following exposure to MgAl_2_O_4_-S-NPs at different concentrations. The nanoparticles exhibited both bacteriostatic and bactericidal effects, with activity being demonstrated against the tested bacterial strains. Mechanochemically synthesized MgAl_2_O_4_-S-NPs are promising candidates for biomedical applications, including dental materials, antimicrobial coatings, and infection-control strategies. Overall, the findings highlight the potential of MgAl_2_O_4_-S-NPs as effective antimicrobial agents that can be produced through an environmentally friendly synthesis route.

## 1. Introduction

Magnesium aluminate (MgAl_2_O_4_) spinel nanostructures exhibit promising properties for dental applications. These nanoceramics demonstrate excellent chemical, thermal, mechanical, and optical characteristics [1]. MgAl_2_O_4_ spinel scaffolds, when combined with hydroxyapatite nanorods, show potential for biomedical use due to their biocompatibility and structural integrity [2]. Nanostructured MgAl_2_O_4_ spinel can be fabricated with a uniform grain size distribution, resulting in high room-temperature strength, improved erosion resistance, and near-theoretical in-line IR transmission [3]. Transparent MgAl_2_O_4_ spinel nanoceramics can be produced at relatively low temperatures (500–700 °C) under high pressure, yielding average grain sizes below 100 nm and high transparency, despite relative densities below 99% [4]. These properties make MgAl_2_O_4_ spinel nanostructures suitable for various applications, including optically transparent windows and armors. With respect to their antibacterial properties, there is limited information available. Recently, a study reported the synthesis of MgO/MgAl_2_O_4_ nanoparticles using a hydrothermal technique and evaluated their activity against four Gram-positive strains—*Staphylococcus epidermidis*, *Staphylococcus aureus*, *Micrococcus luteus*, and *Bacillus subtilis*—and four Gram-negative strains: *Serratia marcescens*, *Escherichia coli*, *Pseudomonas aeruginosa*, and *Klebsiella pneumoniae* [5]. However, there are no reports about their antibacterial properties and potential use against *Enterococcus faecalis* and *Helicobacter pylori*, commensal pathogenic species associated with severe human infections [6].

It is known that magnesium-based nanostructures exert antimicrobial effects that may support dental caries prevention [7]. Zein-coated magnesium oxide nanoparticles, tested via Kirby–Bauer and direct contact methods at concentrations of 1% and 2%, have been shown to inhibit *S. aureus*, *Streptococcus mutans*, *E. faecalis*, and *Candida albicans* [7]. Magnesium-doped hydroxyapatite (at doses from 0.009 to 5 mg/mL) reduced biofilm formation by *P. aeruginosa* and *S. aureus* and moderately suppressed *C. albicans*, while a PMMA/hydroxyapatite/magnesium oxide nanocomposite yielded inhibition zones of 1 to 5 mm against *S. mutans* [8]. Mechanisms of action include metal ion release, oxidative stress induction, and disruption of bacterial membranes; one report also noted improved mineral content and mechanical properties in dental adhesives upon nanoparticle incorporation [9,10]. Also, they have been reported to inhibit oral bacterial growth and promote osteoblast viability, indicating their dual functionality in dental applications [11,12]. Although none of the studies directly evaluated MgAl_2_O_4_ nanostructures, the antimicrobial outcomes of closely related magnesium-based systems suggest that such formulations hold promise for reducing oral bacterial activity in dental materials.

The purpose of this work is to demonstrate the potential antibacterial properties against *Enterococcus faecalis* and *Helicobacter pylori* of the mechanochemically synthesized MgAl_2_O_4_ spinel nanostructures.

## 2. Results

### 2.1. Synthesis and Characterization of MgAl_2_O_4_/S-NPs

**Figure 1:**
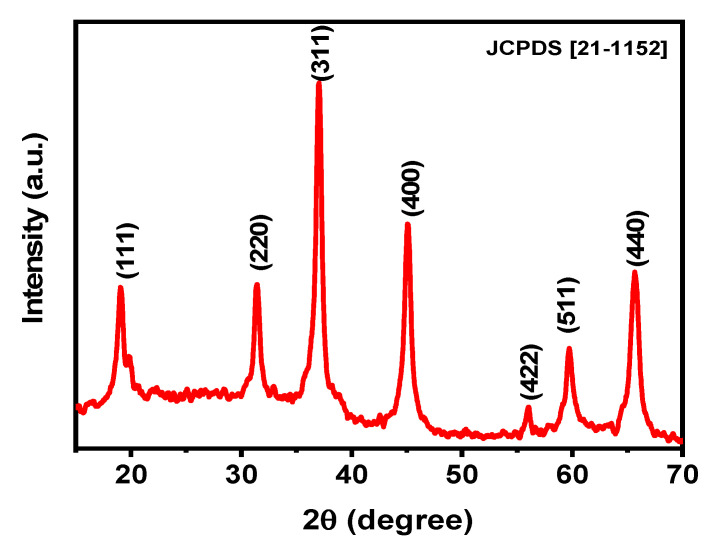
XRD pattern showing peaks at 2θ values of 19.0 (111), 31.5 (220), 37.0 (311), 45.1 (400), 56.0 (422), 59.7 (511), and 65.6 (440), corresponding to the cubic spinel structure of MgAl_2_O_4_ (JCPDS No. 075-1796). No additional reflections associated with possible intermediate phases such as Al_2_O_3_ or MgO were detected, supporting the formation of single-phase MgAl_2_O_4_/S-NPs.

Using the mechanosynthesis approach, MgAl_2_O_4_/S-NPs were obtained with the below characteristics. The X-ray diffraction (XRD) pattern is depicted in Figure 1 the peaks in 2θ (°) values at 19.0 (111), 31.5 (220), 37.0 (311), 45.1 (400), 56.0 (422), 59.7 (511), and 65.6 (440) can be accurately indexed to cubic spinel-structured MgAl_2_O_4_ (JCPDS File No. 075-1796). Other peaks that could indicate the presence of intermediate products, such as Al_2_O_3_ and MgO, have not been observed in the pattern, confirming the single-phase nature of the as-prepared MgAl_2_O_4_/S-NPs. Furthermore, the average crystallite size of MgAl_2_O_4_ was estimated using the Scherrer equation to be 11 ± 2 nm.

**Figure 2:**
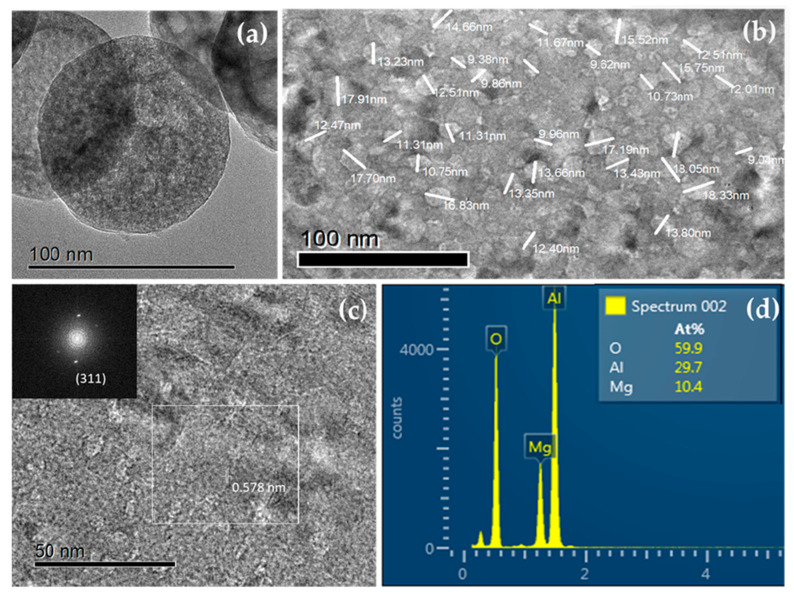
(**a**,**b**) High-resolution transmission electron microscopy (HR-TEM) images of MgAl_2_O_4_/S-NPs revealing polyhedral nanoparticles exhibiting slight agglomeration and measuring under 100 nm. (**c**) The measured interplanar spacings correspond to the characteristic reflections of the MgAl_2_O_4_ crystal structure. (**d**) Energy-dispersive X-ray spectroscopy (EDS) confirming the presence of magnesium (Mg), aluminum (Al), and oxygen (O), with no additional elements being detected.

High-resolution transmission electron microscopy (HR-TEM) micrographs provided additional information, showing polyhedral nanoparticles with some degree of agglomeration and sizes below 100 nm. Energy-dispersive X-ray spectroscopy (EDS) showed the presence of magnesium (Mg), aluminum (Al), and oxygen (O), with no other elements being detected. The interplanar distances coincide with the reflections of the crystal structure of the MgAl_2_O_4_ (Figure 2a–d).

### 2.2. Antibacterial Test

**Figure 3:**
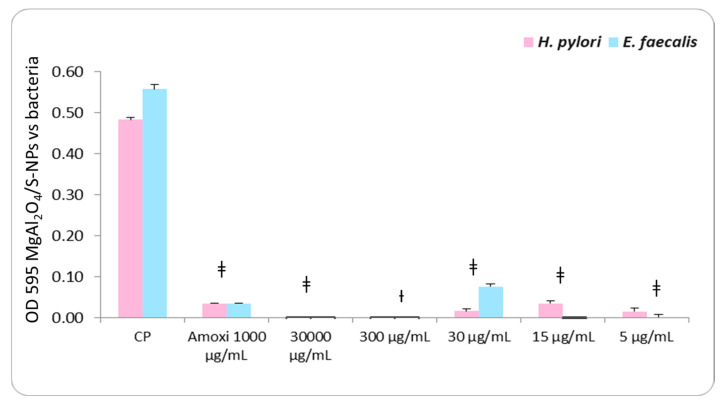
Graph of the broth microdilution assay showing the OD_595_nm values of *Helicobacter pylori* (*H. pylori*) and *Enterococcus faecalis* (*E. faecalis*). † indicates *p* < 0.01, and ‡ indicates *p* < 0.001.

Both *H. pylori* and *E. faecalis* strains exhibited robust positive growth, confirming their suitability as positive controls (+) for the antimicrobial susceptibility assays. For *H. pylori*, susceptibility to MgAl_2_O_4_ nanoparticles was observed at concentrations of 30,000, 30, 15, and 5 µg/mL, with statistically significant differences (*p <* 0.001) relative to the positive control, except at 300 µg/mL, where no significant difference was detected (Figure 3, Table 1). Similarly, *E. faecalis* demonstrated susceptibility at 30,000, 30, 15, and 5 µg/mL (*p <* 0.001), and at 300 µg/mL (*p <* 0.01), when compared with the positive control (Figure 3, Table 1).

**Figure 4:**
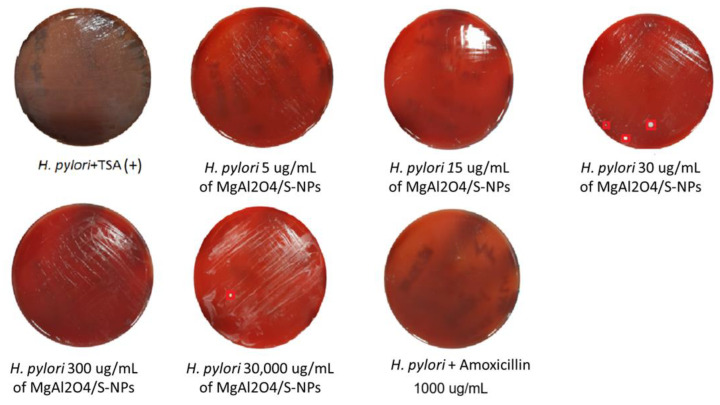
Results of *H. pylori* cell viability at different concentrations of MgAl_2_O_4_/S-NPs. It can be observed that at concentrations of 30 and 30,000 µg/ mL, there was a presence of viable bacteria (CFU).

**Figure 5:**
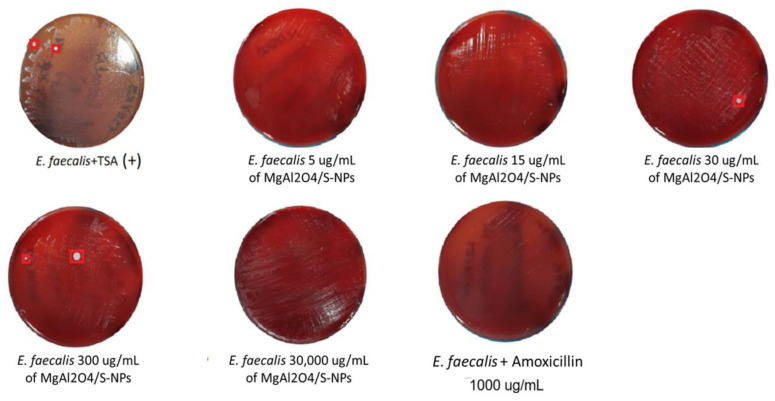
Results of *E. faecalis* cell viability at different concentrations of MgAl_2_O_4_/S-NPs. It can be observed that at concentrations of 30 and 30,000 µg/ mL, there was a presence of viable bacteria and CFU. TSA: Trypticase Soya Agar.

Cell viability recovery outcomes are shown in Figure 4 and Figure 5 at 30,000 µg/mL. Both bacterial strains exhibited positive growth; however, this concentration presented a marked reduction in OD_595_ nm values relative to the positive control (Figure 3 and Table 1).

At 300 µg/mL, *H. pylori* still demonstrated positive growth, whereas *E. faecalis* exhibited only two CFU. At 30 µg/mL, recovery decreased to three CFU for *H. pylori* and one CFU for *E. faecalis*. This is reason to consider 30 µg/mL as the minimum inhibitory concentration, with more than 90% of inhibition cells for *H. pylori* (0.483 − 0.0163/0.483 × 100 = 96.62%) and an inhibitory concentration higher than 90% MIC_90_ for *E. faecalis* (0.558 − 0.076 /0.558 × 100 = 86.37%); both species exhibited bacteriostatic properties at the 30 µg/mL concentration by the countable recovery of colonies.

At 15 µg/mL, no CFU growth was detected for either strain. At the 5 µg/mL concentration, *H. pylori* still demonstrated uncountable positive growth in CFU, whereas *E. faecalis* exhibited no growth in CFU, indicating strong bactericidal activity at the low concentration. With the last results, we considered 15 µg/mL as the minimum bactericidal concentration for *H. pylori* and 5 µg/mL for *E. faecalis,* with 96.06% (0.483 − 0.015/0.483 × 100) and 96.89% (0 − 0.558/0.558 × 100) bacterial inhibition, respectively.

Overall, these findings demonstrate that MgAl_2_O_4_/S-NPs exert consistent bacteriostatic and bactericidal activity against both bacteria evaluated. The table of the broth microdilution assay shows the OD_595_ nm values of *H. pylori* and *E. faecalis*. The results represent the mean OD_595_ nm values (±standard error of the mean) obtained from triplicate assays, compared pairwise with the average positive growth controls (10^4^ cells) using Student’s *t*-test (Sig. t-S). Abbreviations: SE, standard error of the mean; CFU, colony-forming unit.

## 3. Discussion

The characteristics of the MgAl_2_O_4_/S-NPs that were synthesized in this study using a solvent-free, environmentally friendly mechanochemical method are consistent with findings from other studies that have reported the formation of MgAl_2_O_4_ spinel crystallographic phases, although variations in particle size and synthesis methods have been observed [5,13,14,15,16].

The mechanochemically synthesized MgAl_2_O_4_ spinel nanoparticles (MgAl_2_O_4_/S-NPs) exhibit effective bacteriostatic and bactericidal activity against *H. pylori* and *E. faecalis* at relatively low concentrations (MIC = 30 µg/mL; MBC from 5–15 µg/mL). This result may be attributed to the intrinsic characteristics of the bacterial strains used in this study. *H. pylori* is a Gram-negative microaerophilic bacterium with a cell envelope composed of an inner membrane, a periplasmic peptidoglycan layer, and an outer membrane consisting of lipopolysaccharides and phospholipids [17]. In contrast, *E. faecalis* is a Gram-positive facultative aerobic bacterium with a cytoplasmic lipid membrane surrounded by a thick cell wall composed of peptidoglycan, teichoic acid, and a surface polysaccharide antigen [18,19].

It can be inferred that the electrostatic nature of the *H. pylori* cell wall makes it more susceptible to the penetration of MgAl_2_O_4_/S-NPs through its envelope, thereby enhancing their bacteriostatic activity at lower concentrations [20]. At higher concentrations, however, nanoparticle aggregation may occur, forming nanoclusters that adhere irregularly to the bacterial surface and hinder intracellular penetration, as previously reported in the literature [20,21,22].

Although not directly focused on MgAl_2_O_4_, other studies have reported antimicrobial effects of metallic and metal oxide nanoparticles, such as silver, gold, copper, and zinc oxide, that are consistent with the present findings. The size, shape, and concentration of nanoparticles strongly influence their interaction with bacterial membranes, playing a key role in their antimicrobial performance [23,24].

For example, green-synthesized ZnO nanoparticles have been shown to exert potent antibacterial and antibiofilm activity against multidrug-resistant *E. coli*, highlighting the ability of metal oxide nanostructures to disrupt bacterial growth through mechanisms likely involving surface defects and reactive oxygen species generation (ZnO-NPs using *Stevia rebaudiana* as a reducing/capping agent) [25]. Likewise, composite oxide nanoparticles such as MgO, ZnO, and chitosan hydroxyapatite systems have demonstrated effectiveness against carbapenem-resistant Gram-negative clinical isolates, underscoring that mixed oxide systems can be active against both Gram-negative and Gram-positive bacteria when appropriately engineered [26].

In the context of dental materials, MgO nanoparticles obtained by mechanosynthesis have been evaluated for antibacterial activity against *S. aureus*, *E. faecalis*, and *E. coli*, as well as for compatibility with osteoblast cells, indicating that magnesium-based nanomaterials can combine antimicrobial functionality with potential biocompatibility, which is relevant to clinical use [12]. Although the specific bacterial strains differ, these results support the broader observation that magnesium-containing oxide nanoparticles can exert antibacterial effects across multiple pathogens.

Direct comparisons with other MgAl_2_O_4_ systems are also informative. Hydrothermally synthesized MgAl_2_O_4_ nanoparticles with sizes in a similar range (~12 nm) have been reported to exhibit antibacterial activity against *S. aureus*, although specific quantitative comparisons (MIC/MBC) are not universally provided in the literature [14]. Rare-earth-doped MgAl_2_O_4_:Tb^3+^ nanophosphors synthesized by sol–gel methods were found to show antimicrobial activity against *E. coli*, *S. aureus*, and *C. albicans*, with inhibitory effects increasing at higher dopant concentrations, suggesting that modifications to spinel composition can enhance antimicrobial potency, likely through their altered surface chemistry and electronic structure, which influence interaction with microbial cells [27]. For instance, a recent study reported the eco-friendly synthesis of MgO/MgAl_2_O_4_ core/shell nanostructures via a hydrothermal route and evaluated their activity against a broader panel of Gram-positive and Gram-negative bacterial strains, confirming that these nanoparticles exhibited antibacterial effects across diverse pathogens. The authors reported particle sizes predominantly in the range of 90–150 nm and concluded that these were well-optimized for antimicrobial performance, underscoring that nanoscale structural properties and surface area play critical roles in antibacterial efficacy [5].

These comparative findings highlight that antibacterial activity in metal oxide and spinel nanoparticles correlates with phase purity, defect chemistry, particle size, and surface properties rather than with the mere presence of a specific crystal structure. In MgAl_2_O_4_/S-NPs, the small crystallite size (~11 nm) and polydispersity observed by HR-TEM likely contribute to a high surface area and an increased density of surface defect sites, which could facilitate electron transfer, surface redox reactions, and the generation of reactive oxygen species (ROS).

These previously reported nanoparticles share a mechanism of action that is similar to MgAl_2_O_4_ NPs, mainly through the generation of reactive oxygen species (ROS), which are considered a major contributor to antibacterial activity in clinical dentistry [9,20,23,24].

Considering potential factors that could facilitate reactive oxygen species (ROS) production in nanostructured oxide materials, recent studies on doped and defect-rich magnesium oxide systems provide valuable mechanistic insight. For instance, Yin et al. demonstrated that microwave-assisted green synthesis of K-doped MgO nanoparticles leads to a high density of surface oxygen vacancies, which promote ROS generation and enhance antibacterial performance against *E. coli* and *S. aureus*, thereby directly linking defect sites to ROS-mediated biocidal effects [28]. Similarly, investigations of Li-doped MgO have shown that synthesis parameters such as fuel type and dopant incorporation significantly influence microstructure and surface defect density, implying a central role of defect-mediated ROS mechanisms in antibacterial activity [29]. In parallel, studies on oxide spinel materials emphasize the strong influence of composition and defect structure on electronic states and surface reactivity. First-principles analyses of composition-dependent spinel systems, such as ZnO·nAl_2_O_3_, reveal that slight deviations from the ideal cation distribution and compositional variations markedly alter local bonding environments and electronic structure [30], while experimental evaluations of composition-dependent thermophysical and mechanical properties further underscore the sensitivity of defect populations to compositional changes in related spinel systems [31]. In the context of mechanochemically synthesized MgAl_2_O_4_ spinel nanoparticles, trace impurities originating from precursors or milling media—although below the detection limits of XRD—may introduce localized energy states, while oxygen non-stoichiometry and compositional variations suggested by EDS data can generate oxygen vacancies and cation disorder within the lattice. Together, these defect-related features act as active sites for molecular oxygen activation and electron transfer, thereby enhancing ROS formation and plausibly contributing to the observed antibacterial behavior.

*H. pylori* has been shown to be susceptible to antibiotics in vitro but often exhibits resistance in vivo, which is believed to result from limited drug penetration to the infection site at effective antimicrobial concentrations [32]. This bacterium has been reported to develop resistance to nitroimidazoles, macrolides, quinolones, and rifamycins [32]. Similarly, *E. faecalis* is recognized as one of the main causative agents of nosocomial infections and has shown resistance to multiple antibiotics, including ampicillin, gentamicin, streptomycin, ciprofloxacin, levofloxacin, erythromycin, vancomycin, minocycline, and tetracyclines [33].

The literature suggests that nanoparticles may enhance antibiotic efficacy by altering bacterial cell wall permeability in both *H. pylori* and *E. faecalis*. By disrupting the structural integrity of their cell envelopes, these microorganisms become more susceptible to conventional antimicrobials [32,33].

Nanoparticles have broad applications in dentistry, where they have been incorporated into restorative materials such as adhesives and composite resins to prevent secondary caries and biofilm formation. Examples include mineral trioxide aggregate (MTA) and polymethyl methacrylate (PMMA) modified with nanoparticles, which have been used in the fabrication of complete dentures [34]. In endodontics, nanoparticles could serve as irrigant agents or as coatings for gutta-percha cones used in root canal obturation, improving disinfection efficiency [34].

Overall, the results of this study are consistent with increasing evidence showing that nanostructured metal oxides, including mechanochemically synthesized MgAl_2_O_4_ nanoparticles, can exhibit pronounced antibacterial activity at relatively low concentrations, with effectiveness that is comparable to or greater than those reported for other oxide-based nanomaterials. The antibacterial behavior observed herein is likely associated with a synergistic interplay between a high specific surface area, enhanced surface reactivity, and defect-mediated reactive oxygen species (ROS) generation, which are intrinsic to the nanostructured spinel framework, rather than ion release or bulk toxicity mechanisms.

These findings highlight the potential of MgAl_2_O_4_ nanoparticles for application in dental and clinical biomaterials, particularly considering their efficacy at the lowest evaluated concentrations, which may support cost-effective implementation. However, further investigations are required to assess their activity against antibiotic-resistant bacteria, such as methicillin-resistant *Staphylococcus aureus* (MRSA), as well as against biofilm-forming microorganisms [35]. In addition, comprehensive in vivo and cellular-level studies are necessary to evaluate cytotoxicity and long-term biological interactions, ensuring the safety and translational viability of these nanomaterials for biomedical applications.

## 4. Materials and Methods

### 4.1. Synthesis and Characterization of MgAl_2_O_4_/S-NPs

The MgAl_2_O_4_ nanoparticles used in this study were synthesized via a mechanochemical route based on high-energy and continuous milling of precursor oxides. Commercial magnesium oxide (MgO) and aluminum oxide (Al_2_O_3_) were used as starting materials and mixed in a stoichiometric molar ratio of 1:1. Prior to combined milling, each oxide was ground separately in an agate mortar for approximately 20 min, or until no further observable changes in particle texture were detected, to ensure homogenization and particle size reduction. Subsequently, the MgO and Al_2_O_3_ powders were combined and milled together for an additional 20 min to promote intimate contact and solid-state diffusion between the reactants. The resulting powder mixture was then subjected to thermal treatment in a conventional oven at 800 °C for 2 h to facilitate the formation of the MgAl_2_O_4_ spinel phase. After calcination, the product was washed sequentially 3 times with deionized water and acetone to remove residual impurities and loosely bound species, followed by drying prior to characterization. The formation of nano-MgAl_2_O_4_ can be described by the following solid-state reaction:

$${\mathrm{MgO}+\mathrm{Al}}_{2}O_{3}\overset{+800\;{}^{\circ}C}{\underset{\mathrm{Milling}}{\to }}{\mathrm{MgAl}}_{2}O_{4}$$

The resulting powders were calcined at 800 °C for 2 h, followed by sequential washing with distilled water and acetone. After each washing step, the product was separated by centrifugation at 3000 rpm.

For the characterization of the synthesized nanoparticles, transmission electron microscopy (TEM) and X-ray diffraction (XRD) were employed to determine the morphology, crystalline structure, and chemical bonding characteristics, respectively.

### 4.2. Antibacterial Test

#### 4.2.1. Bacterial Strains and Culture Conditions

Lyophilized strains of *H. pylori* (ATCC 43629) and *E. faecalis* (ATCC 51299) were used in this study. The strains were rehydrated using *Mycoplasma* broth medium and subsequently cultured on Trypticase Soy Agar (TSA) plates to assess purity and cell viability. A total of 40 TSA Petri dishes were prepared for these preliminary evaluations.

#### 4.2.2. Preparation of Antibiotic Stock Solutions

The antibiotic stock solutions were prepared using amoxicillin at a 1 mg/mL concentration. It was dissolved in sterile water in a 125 mL screw-cap Erlenmeyer flask. Once fully dissolved, the solutions were sterilized by filtration using 0.2 μm syringe filters and aliquoted into 2 mL cryovials. The aliquots were stored at −20 °C until use in the broth microdilution assays.

#### 4.2.3. Preparation of Bacterial Inocula

After confirming bacterial growth on TSA plates, colonies were gently scraped and suspended in Trypticase Soy Broth (TSB). Each 1.8 mL microdilution tube was filled with 1 mL of the suspension, and the first tube (“OD_1_”) was used for optical density calibration. The optical density (OD) measured at 600 nm corresponded to an approximate concentration of 10^9^ CFU/mL.

Two sets of sterile 1.8 mL microdilution tubes were prepared and labeled from 1 to 5, with an additional tube containing only medium serving as the negative control for each strain. Serial dilutions were performed to obtain a final concentration of 10^4^ CFU/mL. To ensure homogeneous dispersion of bacterial cells, brief intermittent sonication (<2 s) was applied, with sterilization of the probe before each use.

For the 10^8^ CFU/mL dilution, 0.9 mL of TSB was mixed with 0.1 mL of the OD_1_ suspension. This procedure was repeated to obtain subsequent dilutions down to 10^4^ CFU/mL, ensuring sufficient volume for the microdilution assays.

#### 4.2.4. Preparation and Dilution of MgAl_2_O_4_/S-NPs

To evaluate the antimicrobial activity of MgAl_2_O_4_/S-NPs, five sterile 1.8 mL microdilution tubes were prepared for each bacterial strain and labeled from 1 to 5. Nanoparticle suspensions were prepared at concentrations ranging from 30 mg/mL to 0.005 mg/mL using molecular-grade water as the diluent. All procedures were performed under aseptic conditions.

#### 4.2.5. Antimicrobial Susceptibility Assay (Broth Microdilution Method)

The antimicrobial activity of MgAl_2_O_4_/S-NPs was evaluated using the broth microdilution method in sterile 96-well microplates. A volume of 100 μL of the amoxicillin solution (1 mg/mL) was added to columns 6 and 12, specifically in rows A, C, E, G, and H, to serve as antibiotic controls. Subsequently, 100 μL of the *H. pylori* suspension (10^4^ CFU/mL) was added to wells in columns 1–6 (rows A, C, and E), whereas 100 μL of the *E. faecalis* suspension was added to wells in columns 7–12 (rows A, C, and E).

All pipetting procedures were performed under sterile conditions using a Bunsen burner to maintain aseptic technique. The microplates were sealed with parafilm and incubated at 37 °C for 48 h under aerobic conditions with gentle agitation at 80 rpm. Each experimental well was performed in triplicate.

#### 4.2.6. Recovery and Assessment of Cell Viability

After 48 h of incubation, the microplates were analyzed using a FilterMax F5 microplate reader (Molecular Devices, LLC. 3860N.First Street, San Jose, CA, USA) at 595 nm to assess bacterial growth. From each assay, a 100 μL aliquot was collected from the first triplicate well and cultured on TSA plates to observe bacterial growth viability after 42 h of incubation.

### 4.3. Data Analysis

OD values at 595 nm were averaged from triplicates, and the standard error of the mean per measurement was calculated and statistically compared with the corresponding positive growth controls for each strain (10^4^ CFU/mL). Obtained OD values were analyzed using a normalized Shapiro–Wilk test to assess the normality of the distribution. Statistical analyses were performed using the parametric Student’s *t*-test with a 95% confidence interval (CI), employing IBM SPSS Statistics v21 (IBM Corp., Armonk, NY, USA).

Agar viability and colony-forming units (CFU) were manually counted using a manual colony counter and a stereomicroscope. Results were used to determine the Minimum Inhibitory Concentration (MIC), defined as an OD reduction with statistical differences and a visible reduction in bacterial growth with countable colonies of the recovery of viability. The Minimum Bactericidal Concentration (MBC) was defined as an OD reduction with statistical differences, and a complete absence of CFU growth was interpreted as bactericidal activity. For the interpretation of breakpoints of antibacterial inhibition at the 50 and 90% percentile (MIC_50_, MIC_90_), ODs were calculated using the following equation: OD control − OD sample /OD control × 100 to obtain % of bacterial inhibition.

## 5. Conclusions

In the present study, MgAl_2_O_4_/S-NPs synthesized via a mechanochemical method demonstrated a bacteriostatic effect at the tested concentrations against two antibiotic-resistant bacterial strains of dental and medical relevance. These findings provide evidence supporting the potential use of MgAl_2_O_4_/S-NPs in the development of new therapeutic strategies against multidrug-resistant pathogens in dental and medical practice, complementing the antibacterial behavior observed in microbiological assays.

Further research is recommended to investigate the application of MgAl_2_O_4_/S-NPs under varied mechanosynthesis parameters to obtain different particle sizes, as well as to evaluate a broader range of concentrations and exposure times. Additional studies incorporating combinatory approaches and in vivo cytotoxicity assessments are also required to confirm their efficacy and potential applicability in medical and dental treatments.

## Figures and Tables

**Table 1:** Values of *Helicobacter pylori* (*H. pylori*) and *Enterococcus faecalis* (*E. faecalis*).

***H. pylori***	**Media**	**SEM**	**Sig. t-S**	**Agar Viability CFU**
C (-) *H. p*	0.483	0.006	0.6039	(+)
Amoxi 1000 µg/mL + H. p	0.035	0	0.0005	(-)
MgAl_2_O_4_/S-NPs 30,000 µg/mL	0	0	0.0004	(+)
MgAl_2_O_4_/S-NPs 300 µg/mL	0	0	0.0549	(+)
MgAl_2_O_4_/S-NPs 30 µg/mL	0.016	0.004	0.0005	**3**
MgAl_2_O_4_/S-NPs 15 µg/mL	0.034	0.007	0.0005	(-)
MgAl_2_O_4_/S-NPs 5 µg/mL	0.015	0.008	0.0005	(+)
***E. faecalis***	**Media**	**SEM**	**Sig. t-S**	**Agar Viability CFU**
C (-) *E. f*	0.558	0.011	0.0704	(+)
Amoxi 1000 µg/mL + E. f	0.046	0	0.0002	(-)
MgAl_2_O_4_/S-NPs 30,000 µg/mL	0	0.019	0.0000	(+)
MgAl_2_O_4_/S-NPs 300 µg/mL	0	0	0.0081	2
MgAl_2_O_4_/S-NPs 30 µg/mL	0.076	0.005	0.0003	**1**
MgAl_2_O_4_/S-NPs 15 µg/mL	0	0	0.0001	(-)
MgAl_2_O_4_/S-NPs 5 µg/mL	0	0.007	0.0003	(-)

## Data Availability

The data presented in this study are available upon request from the corresponding author.
